# Role of miRNA-146 in proliferation and differentiation of mouse neural stem cells

**DOI:** 10.1042/BSR20150088

**Published:** 2015-09-10

**Authors:** Wei-Zhong Xiao, An-Qing Lu, Xin-Wei Liu, Zhe Li, Ying Zi, Zhi-Wei Wang

**Affiliations:** * Department of Neurology, Shanghai Pudong Hospital, Fudan University, Shanghai 201399, PR China; † Department of Orthopedics, Changhai Hospital, Second Military Medical University, Shanghai 200433, PR China; ‡ Department of Neurosurgery GanSu Provincial Hospital, LanZhou, GanSu 730000, PR China; § Department of Orthopedics, General Hospital of Shenyang Military Area Command of Chinese PLA, Shenyang, Liaoning 110016. PR China; ║ No.705 Hospital of Chinese Airforce, Shenyang 110000, PR China; ¶ Department of Emergency, 463rd Hospital of PLA, Shenyang 110042, PR China

**Keywords:** cell proliferation, differentiation, microRNA, neural stem cells

## Abstract

Neural stem cells (NSCs) have been defined as neural cells with the potential to self-renew and eventually generate all cell types of the nervous system. NSCs serve as an ideal cell type for nervous system repair. In the present study, *miR-146* overexpression and predicted target (notch 1) were used to study proliferation and differentiation of mouse NSCs. shRNA were used to demonstrate the function of Notch 1 in proliferation of mouse NSCs and luciferase reporter assay was used to assess and confirm the binding sequence of 3′-UTR between Notch 1 and *miR-146*. Results showed that *miR-146* overexpression and knockdown of notch 1 inhibited proliferation of mouse NSCs under serum-free cultural conditions and promoted spontaneous differentiation of mouse NSCs under contained serum cultural conditions respectively. Mouse NSCs spontaneously underwent differentiation into neurogenic cells with contained serum medium. However, when *miR-146* was overexpressed, differentiation efficiency of glial cells from NSCs was increased, suggesting that Notch1 promoted NSC proliferation and repressed spontaneous differentiation of NSC in serum-free medium. In conclusion, our results demonstrate that *miR-146* promoted spontaneous differentiation of NSCs, and this mechanism was influenced by *miR-146*, as well as its target (notch 1) and downstream gene.

## Abstract (precis)

In the present study, *miR-146* promoted spontaneous differentiation of neural stem cells (NSCs), and this mechanism was influenced by *miR-146*, as well as its target (notch 1) and downstream gene.

## INTRODUCTION

miRNAs are a fundamental class of biological molecules that play a crucial role in development [1]. miRNA dysfunction has been linked to cancer [2], as well as other biological processes. Genes encoding miRNAs, which are found in most eukaryotes, produce short (18–25 nt) RNAs that bind to mRNA transcripts and down-regulate expression either through mRNA destabilization or translational repression [3,4]. miRNAs regulate gene expression by guiding the RNA-induced silencing complex to a target sequence, which is usually located at the 3′-UTR of mRNAs [5]. The widespread involvement of miRNAs in regulating developmental processes, physiological responses and pathological conditions in animals has been amply demonstrated [4,6]. Nonetheless, the specific functions of each miRNA in the various contexts in which it is expressed are only beginning to be discovered. Neural stem cells (NSCs) have the ability to self-renew and are capable of differentiating into neurons, astrocytes, and oligodendrocytes. NSCs have been isolated from the developing brain and more recently from the adult central nervous system. In recent years, a variety of signal pathways have been identified that ensure NSC maintenance and control the development of neural tissues and progenitor cells. Thus, miRNAs are potentially key post-transcriptional regulators in NSCs self-renewal and differentiation. Distinct sets of miRNAs have been shown to be specifically expressed in embryonic stem cells [7,8]. Knockout or knockdown of Dicer causes embryonic lethality and loss of stem cell populations [9]. Argonaute family members, which are key components of the RNA-induced silencing complex, are required for maintaining germ line stem cells in various species [10,11]. Together, these observations support a role for miRNAs in stem cell biology. Several brain-specific miRNAs have recently been identified. Among these miRNAs, *miR-146* is a family of miRNA precursors found in mammals, including humans. *miR-146* is primarily involved in the regulation of inflammation and other process that involve the innate immune system [12]. *miR-146* is thought to be a mediator of inflammation, along with another miRNA, *miR-155*. Expression of *miR-146* is up-regulated by inflammatory factors, such as interleukin 1 and tumour necrosis factor-α and *miR-146* dysregulates a number of targets mostly involved in toll-like receptor pathways that bring about a cytokine response as part of the innate immune system [13]. *miR-146* operates in a feedback system or ‘negative regulatory loop’ to finely tune inflammatory responses [14]. Loss of functional *miR-146* could predispose an individual to experience chromosome 5q deletion syndrome [14]. However, the functions of *miR-146* in proliferation and differentiation of NSCs are poorly understood. In the present study, *miR-146* precursor was transfected into NSCs to determine its role in proliferation and differentiation of NSCs.

## MATERIALS AND METHODS

### Isolation and culture of mouse NSCs

NSCs were collected from brain tissue of embryonic BABLC (BALB/cAnNCrl mouse) mice, supplied by the Laboratory Animal Center of Second Military Medical University. Brain tissue was isolated and rinsed three times and then transferred to a Dulbecco's modified Eagles medium (DMEM)/F12 (D/F12; Gibco) medium-containing plate for cultivation into a monoplast suspension after repetitive pipette and mechanical separation. Single cells were plated at 1000 cells/cm^2^ in untreated 25-cm^2^ tissue culture flasks and cultured in serum-free neural medium [D/F12+2% B27 supplement (Gibco)+20 ng/ml endothelial growth factor (EGF) (PeproTech)+bFGF (fibroblast growth factor; PeproTech)]. Medium (50%) was replaced every 2 days.

### Immunofluorescence

Immunofluorescence was used to identify the NSCs in conjunction with specific markers Nestin, Sox 2 (sex determining region Y–box 2) and Pax 6 (paired box protein Pax-6). NSCs were seeded onto 100 μg/ml poly-L-lysine-coated coverslips and allowed to incubate for 24 h. The cells were then fixed with 4% paraformaldehyde for 10 min and washed three times in PBS buffer. The cells were permeabilized with 0.125% Triton X-100 for 10 min and then washed three times in PBS buffer. The cells were then blocked with 10% normal goat serum for 30 min. Mouse NSCs were then incubated with Nestin, Sox 2 and Pax 6 antibodies in a humidified chamber at 4°C overnight. After three PBS buffer washes, the cells were incubated with Cy5.5-labelled secondary antibodies at room temperature for 1 h, the cells were rinsed three times with PBS buffer. Finally, nuclei were labelled by incubating with DAPI (Sigma). The cells were examined using a phase-contrast fluorescence microscope (Olympus).

### Assay of luciferase activity

The 3′-UTR fragments for Notch 1 were generated by PCR using the following primers: 5′-cttggagtgtggtttggttca-3′ and 5′-tcaggctcagagagggcat-3′and cloned into the psiCHECK-2 vector (Promega) downstream from the Renilla luciferase cassette. The predicted *miR-146*-binding site was mutated using the QuikChange II XL Site-Directed Mutagenesis Kit (Stratagene). Human embryonic kidney (HEK)293T cells were grown in a 96-well plate and co-transfected with the luciferase reporter vector together with a miRNA precursor or a negative control (20 nM; Ambion) using Attractene (Qiagen) according to the manufacturer's instructions. Activities of firefly and Renilla luciferase were analysed using the Dual-Luciferase Reporter Assay System (Promega) 24 h after transfection.

### Expression of small RNAs by recombinant lentivirus

The pre-*miR-146* sequence and Notch 1 shRNA were synthesized by Sangon Biotech (Shanghai), cloned into a lentiviral vector and then used to recombine lentivirus in HEK293F cells. The lentivirus, designated as pCMV-SM30, also expressed GFP as a marker for monitoring infection efficiency. Analogous lentivirus expressing only monomeric GFP was used as a control. Real-time PCR was used to evaluate the expression level of *miR-146* after virus infection. miRNAs were isolated from cells using a microRNA isolation kit (Applied Biosystems) according to the manufacturer's instructions. cDNA synthesis was performed using the High-Capacity cDNA Synthesis Kit (Applied Biosystems) with 2 ng of RNA as a template. The miRNA sequence-specific reverse-transcription PCR primers for *miR-146* and endogenous control U6 were purchased from Ambion. Real-time PCR analysis was performed using the Applied Biosystems 7500 real-time PCR system. The gene expression threshold cycle (CT) values of miRNAs from each sample were calculated by normalizing the samples to the internal control U6 and relative quantitation values were plotted.

### Western blotting

Notch 1, the target gene of *miR-146*, was detected by western blot analysis following overexpression of *miR-146* and shRNA. Cells were lysed using M-PER Protein Extraction Reagent (Pierce) supplemented with a protease inhibitor cocktail (PMSF). Protein concentrations of the extracts were measured using the BCA assays (Pierce) and equalized with extraction reagent. Equal amounts of extracts were loaded and subjected to SDS/PAGE, followed by transfer onto nitrocellulose membranes. Specific antibodies and horseradish peroxidase-coupled secondary antibodies were purchased from Santa Cruz. Membranes were probed using ultra-ECL western blotting detection reagents. Glyceraldehyde-3-phosphate dehydrogenase (GAPDH) was used as an internal control.

### Morphological characteristics of NSCs following overexpression of small RNAs

Floating neural spheres was the primary morphology of NSCs *in vitro*. The neural sphere diameter of 1- and 8-day cultures was measured using fluorescence microscope in normal NSCs, *miR-146*-overexpressed NSCs and shRNA-overexpressed NSCs respectively.

### Spontaneous differentiation of NSCs

Spontaneously differentiating cells were divided into three groups: normal NSCs (group A), NSCs after *miR-146* overexpression (group B) and NSCs after shRNA overexpression (group C). The cells were seeded onto plates with D/F12 containing 10% FBS (Gibco). After 10 days, the cells were harvested and neural specific makers (β-III-tubulin and GFAP) were detected and analysed by immunocytochemical staining and flow cytometry. The primary antibodies were β-III-tubulin and GFAP (Glial fibrillary acidic protein, Abcam). The secondary antibodies were conjugated with Cy5.5.

### Real-time PCR

#### Real time PCR

RNA was extracted from cells using Trizol reagent (Invitrogen). Total RNA was reverse transcribed, followed by 30 PCR cycles using RNA PCR kit ver 3.0 (TARAKA). Information of gene specific primer pairs was listed in Table 1. PCR was performed in 50 μl of mixture containing 10 μl of 5× PCR Buffer (TARAKA), 28.5 μl of double distilled water (ddH_2_O), 0.25 μl of Ex-Taq (TARAKA), 0.5μl of forward and reverse primers and 1.5 μl of template cDNA. The cycling conditions consisted of one initial 2-min cycle at 94°C, followed by 30 30-s cycles at 94°C (denaturation), one 30-s cycle at 50°C–60°C (annealing) and one 2-min cycle at 72°C (extension). PCR products were detected by 2.5% agarose gel electrophoresis. Real-time PCR was performed in a 20 μl of mixture containing 10 μl of SYBR premix Ex Taq buffer (Takara), 0.4 μl of fluorescent dye used in PCR (ROX) Reference Dye, 0.8 μM each of forward and reverse primers (Table 1), 1 μl of template cDNA and 7 μl of ddH2O. The cycling conditions consisted of initial 10 s at 95°C followed by 40 cycles of two-temperature cycling: 5 s at 95°C (for denaturation) and 34 s at 60°C (for annealing and polymerization). Each experiment was performed with duplicates in 96-well plate and repeated three times. Gene expression was detected on an ABI 7500 real-time PCR system. The expression level was calculated by the 2-ΔΔCt method to compare the relative expression.

**Table 1:** Primer sequences used in real-time PCR assay

Gene	Gene ID	Primer sequence	*T*_m_ (°C)	Product size (bp)
CBF1	19664	F 5′–AAGATGGCGCCTGTTGTGA-3′	60	109
		R 5′-GCACTGTTTGATCCCCTCGT-3′		
HES 1	15205	F 5′-TGGTCCTGGAATAGTGCTACC-3′	60	212
		R 5′-CTTTCTGTGCTCAGAGGCCG-3′		
HES 5	15208	F 5′-GAGATGCTCAGTCCCAAGGAG-3′	60	203
		R 5′-GCGAAGGCTTTGCTGTGTTT-3′		
GAPDH	14433	F 5′-GGAGAGTGTTTCCTCGTCCC-3′	60	136
		R 5′-ATGAAGGGGTCGTTGATGGC-3′		

## RESULTS

### Bio-characteristics of mouse NSCs

NSCs derived from the dissociation of mouse forebrain samples were suspended in culture medium. The neurospheres were slow to form and the spheres were relatively small. In culture, there were many single cells attached to the flasks. However, as the cells proliferated into small clusters, they detached from the plastic and floated in suspension. Nestin, Sox 2, and Pax 6 protein expressions were detected by immunofluorescence assay. Results demonstrated that different passages of mouse NSCs expressed Nestin, Sox 2 and Pax 6 (Figure 1).

**Figure 1:**
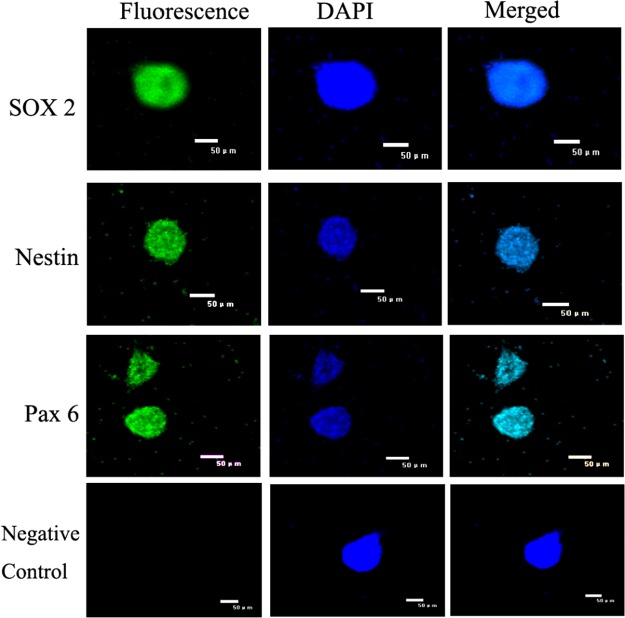
NSC surface marker detection Nestin, Sox 2 and Pax 6 are specific markers for NSCs. Nestin, a protein marker for NSCs, is also expressed in follicle stem cells and their immediate, differentiated progeny. Sox 2 is a transcription factor that is essential for maintaining self-renewal or pluripotency of undifferentiated embryonic stem cells. Sox 2 plays a critical role in the maintenance of NSCs. Pax 6 is a transcription factor present during embryonic development; it is a key regulatory gene for eye and brain development. Within the brain, the protein is involved in development of the NSCs that process smell. The immunofluorescence results showed that mouse NSCs expressed Nestin, Sox 2 and Pax 6. Negative control: PBS was used to replace the primary antibody for immunofluorescence detection.

### Notch 1 is a direct target of miR-146

miRNA target genes are likely to have relatively long and conserved 3′-UTR [4]. We noticed that Notch 1 has a long evolutionarily conserved 3′-UTR, so we used the TargetScan algorithm [15] to search for miRNAs that could potentially regulate Notch 1. The *miR-146* family (*miR-146a* and *miR-146b*) has the same putative target binding sites in Notch 1 in the mouse genome (Figure 2). To directly test whether *miR-146* targets Notch 1, we cloned the 3′-UTRs of Notch 1 downstream of a luciferase reporter, and co-transfected these reporter constructs along with miRNA precursors into the human cell line HEK293T. Co-expression of *miR-146* was found to effectively down-regulate luciferase expression in constructs with these 3′-UTRs (Figure 2). Mutations in the seed sequence of the predicted *miR-146*-binding sites within Notch 1 abolished the inhibitory effects of *miR-146* on luciferase expression. The pre-*miR-146* sequence and Notch 1 shRNA were synthesized and overexpressed in mouse NSCs, expression of *miR-146a/b* was quantified by real-time PCR at 72 h after transfection. As shown in Figure 2(C) and 2(D), *miR-146a/b* levels were significantly elevated after transfection. Protein expression of Notch 1, the putative target gene, was performed on *miR-146a/b*-transfected cells using the ImageJ tools comparative method (Figures 2E and 2F). Additionally, Notch 1 shRNA was assessed in NSCs and results were in accordance with *miR-146* function; Notch 1 protein expression was down-regulated (Figures 2G and 2H).

**Figure 2:**
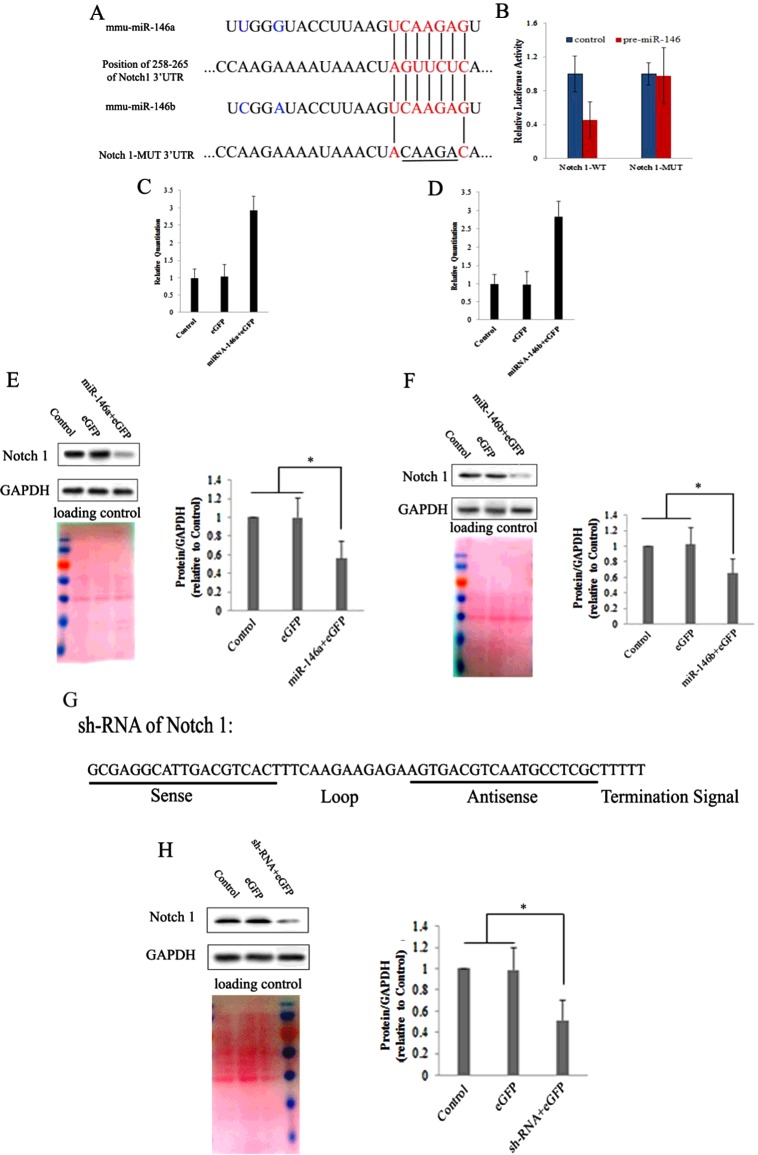
Notch 1 is the direct target of *miR-146* (**A**) *miR-146ab* complementary sites with 3′-UTR of Notch 1. The mutant sequence {Notch 1–MUT (mutation)} is identical to Notch 1–WT (wild type) construct except for five point mutations disrupting base-pairing at the 5′-end of *miR-146* (indicated with a bar). (**B**) Mutating the *miR-146* target site in the 3′-UTR of Notch 1 abolishes inhibition of luciferase activity by endogenous *miR-146* in 293 cells. (**C** and **D**) NSCs were transfected with lentivirus as described in the ‘Materials and Methods’ section and *miR-146* expression was quantified by real-time PCR. (**E** and **F**) Effect of *miR-146ab* on protein expression of Notch 1 and quantification of Notch 1 in NSCs transfected with *miR-146ab*, eGFP or control for 72 h. (**G**) Sequences of shRNAs for Notch 1 genes. (**H**) Western blot analysis of protein levels to confirm siRNA-mediated knockdown of Notch 1. (*P<0.05)

### Role of *miR-146* and shRNA in NSC proliferation

NSC proliferation was assessed according to neural sphere diameter after overexpressed small RNAs (micron, Figure 3) and results demonstrated that NSC proliferation was stagnated. The diameter of neural spheres was measured using fluorescence microscope from 1- to 8-day cultured cells in normal NSCs, *miR-146*-overexpressed NSCs and shRNA-overexpressed NSCs respectively. The curves of neural spheres diameter from 1- to 8-day cultured cells in normal NSCs, *miR-146*-overexpressed NSCs and shRNA-overexpressed NSCs respectively. This result showed a time-dependent increase in neural sphere diameter from normal NSCs. However, the diameter of neural spheres showed no significant difference in NSCs following overexpression of small RNAs. Additionally, gene expression of the Notch pathway showed a time-dependent decrease following overexpression of small RNAs. Moreover, the diameter of neural spheres continuously increased and there was a time-dependent increase in Notch pathway expression in the control group (Figure 4).

**Figure 3:**
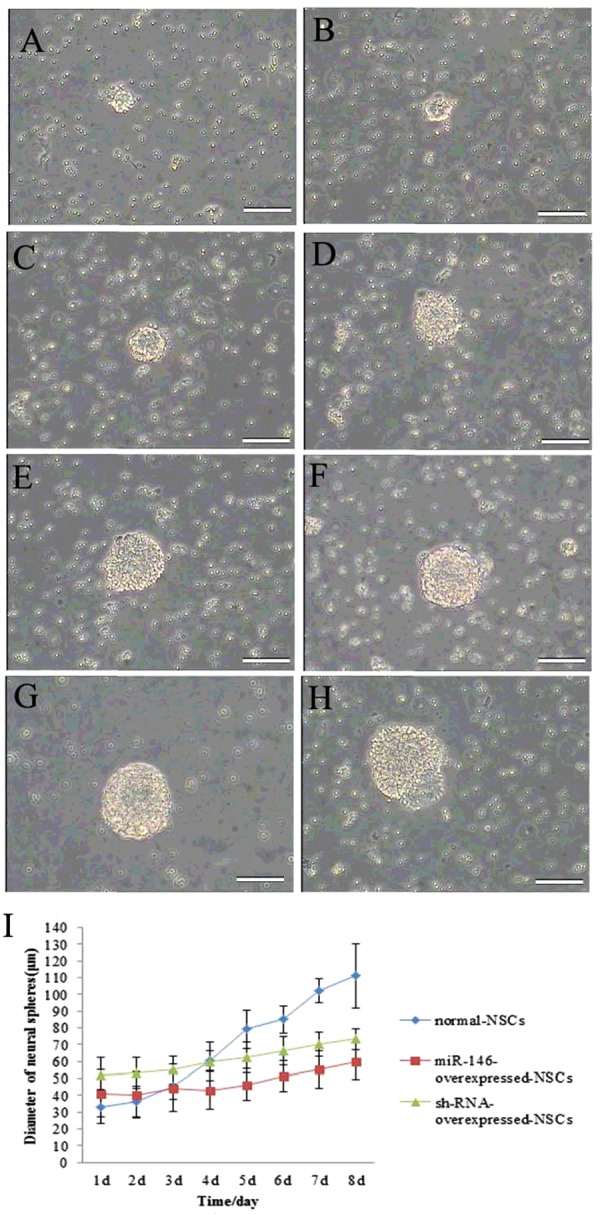
Role of small RNAs in NSC proliferation Floating neural spheres was the primary morphology of NSCs *in vitro*. The diameter of neural spheres was measured using fluorescence microscope from 1- to 8-day cultured cells in normal NSCs, *miR-146*-overexpressed NSCs and shRNA-overexpressed NSCs respectively. (**A**–**H**) NSC proliferation was observed in normal NSCs from 1- to 8-day cultures respectively. (**I**) The curves of neural spheres diameter from 1- to 8-day cultured cells in normal NSCs, *miR-146*-overexpressed NSCs and shRNA-overexpressed NSCs respectively. This result showed a time-dependent increase in neural sphere diameter from normal NSCs. However, the diameter of neural spheres showed no significant difference in NSCs following overexpression of small RNAs.

**Figure 4:**
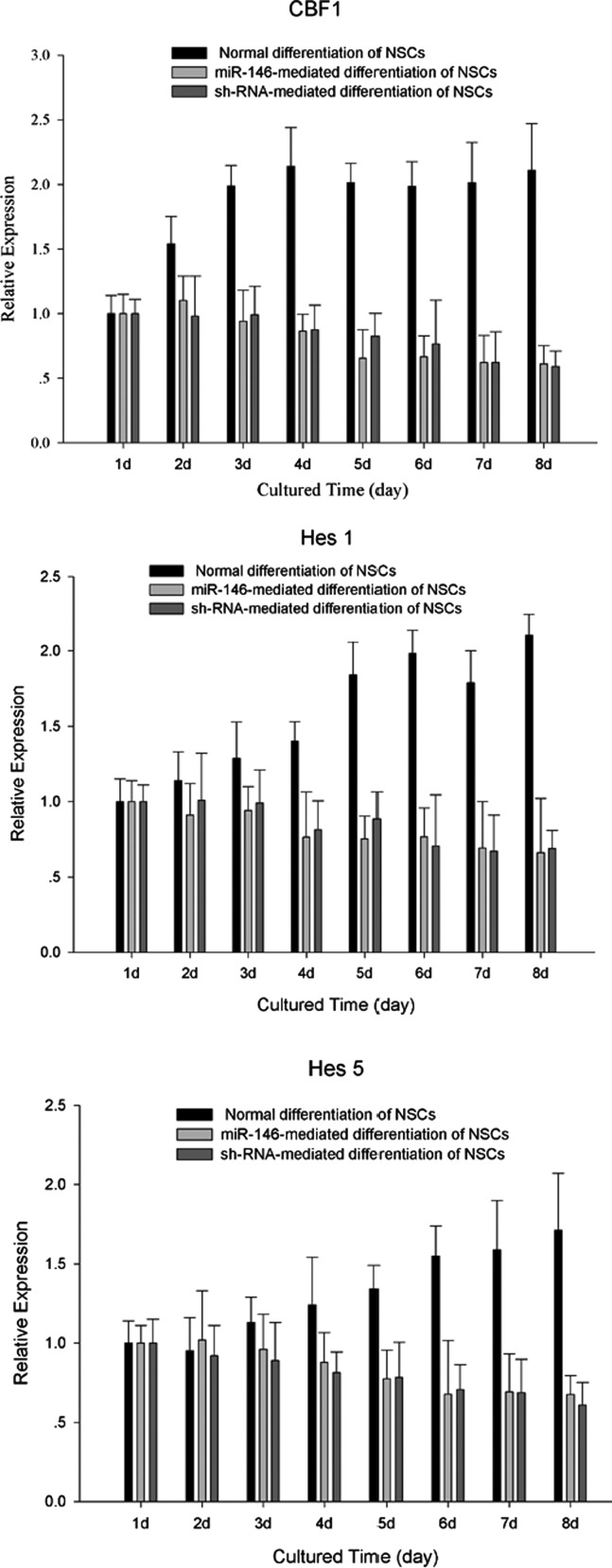
Gene expression of Notch pathway detected in continuously cultured NSCs Gene expressions in each group were determined by real-time PCR and then normalized to that of control (1 day). Data are shown as means ± S.E.M. from three repeated. CBF1, also known as RBP-J, is the human homologue for the *Drosophila* gene suppressor of hairless. Its promoter region is classically used to demonstrate Notch 1 signalling. HES1 influences maintenance of certain stem cells and progenitor cells and is expressed in neuroepithelial cells, radial glial cells and NSCs. Hes1 expression, along with Hes 5, is present in the majority of developing embryos, and is maintained in NSCs expressing Pax 6. Expression of CBF1, Hes 1 and Hes 5 was time-dependent in normal NSCs. However, following overexpression of siRNAs, expression decreased over time.

### Role of *miR-146* and shRNA in spontaneous differentiation of NSCs

NSCs were induced to differentiate into neural cells for 10 days. Following induction, the cells contracted and became round, triangular or cone shaped with multipolar processes. The processes continued to grow with many branches forming and cone-like terminal expansions. A number of cells demonstrated very long processes, which appeared similar to long neuronal axons. After 10 days of induction, immunofluorescence and flow cytometry assay detected β-III-tubulin and GFAP expression (Figure 4).

However, the production of neuronal cells (β-III-tubulin-positive cells) from NSCs after transfection with pre-*miR-146* and shRNA was lower than from normal NSCs. Moreover, transfection of *miR-146* and shRNA dramatically increased the percentage of glial cells (GFAP-positive cells) and reduced the percentage of neuronal cells *in vitro* (Figure 5).

**Figure 5:**
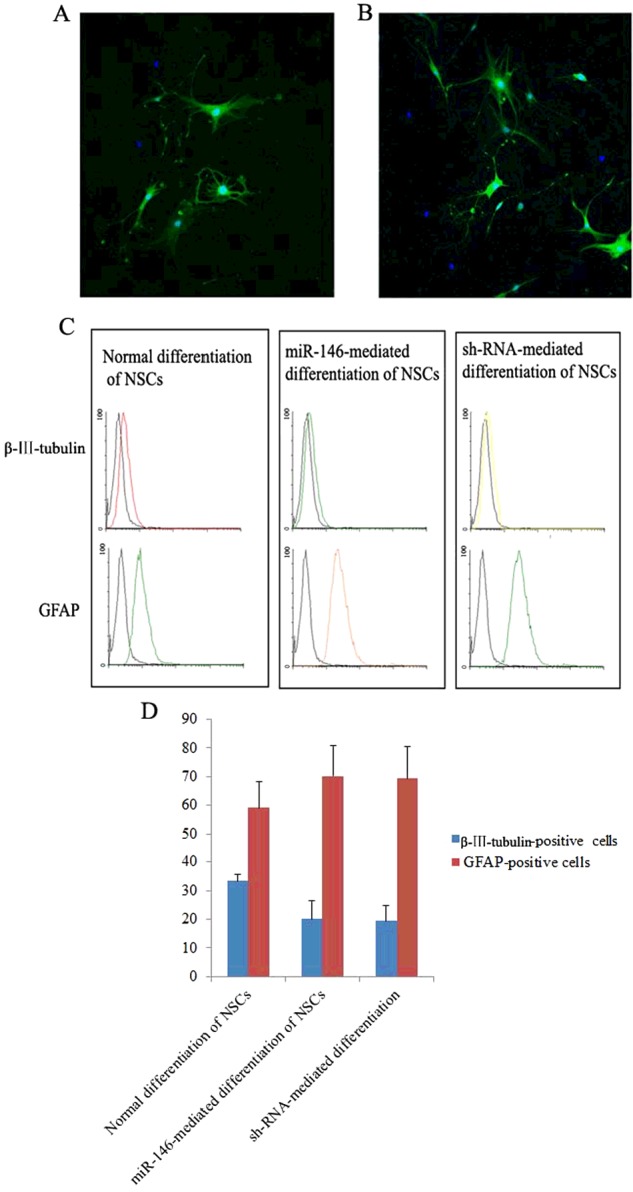
Neural differentiation of NSCs Immunofluorescence staining assay showed expression of neural-specific genes, including β-III-tubulin and GFAP following induction. β-III-tubulin, a specific marker of neurons; GFAP, a specific marker of glial cells. (**A**) GFAP-positive glial cells, (**B**) β-III-tubulin-positive neurons. (**C**) β-III-tubulin and GFAP were analysed in normal differentiation, *miR-146*-mediated differentiation and shRNA-mediated differentiation of NSCs using flow cytometry. (**D**) Percentage of neurons and glial cells assayed from different NSCs, including normal NSCs and siRNA overexpression of NSCs. There is a significant increase in GFAP-positive cells compared with β-III-tubulin-positive cells following siRNA overexpression (*P*<0.01).

## DISCUSSION

In mammals, a wide variety of cells use the Notch signalling system for embryonic development and maintenance of homoeostasis in adults. The role of Notch signalling in neural development has been intensely analysed using conditional inactivation of RBP-J (recombining binding protein suppressor of hairless) and it has been shown that Notch signalling is required for both maintenance of neural stem/progenitor cells and specification of glial cells [16,17]. RBP-J, also known as CBF1, is the human homologue for the *Drosophila* gene suppressor of hairless. Its promoter region is classically used to demonstrate Notch1 signalling [18,19]. Precocious neuronal differentiation observed in Notch pathway-deficient neuro-competent cells is also detected in mammals, such as mice with inactivated Notch 1. A conditional Notch 1 knockout study provides further support that Notch signalling inhibits the premature onset of neurogenesis [20].

miRNAs are small, non-coding RNAs that are cleaved from 70 to 100 nt hairpin pre-miRNA precursors in the cytoplasm by RNaseIII Dicer into their mature form of 19–25 nt. Single-stranded miRNAs bind messenger RNAs of potentially hundreds of genes at the 3′-UTR region with perfect or near-perfect complementarity, resulting in degradation or inhibition of the target messenger RNA [21]. miRNAs are fundamental biological molecules that have been shown to play important roles in biological development [22]. *miR-146a* is a member of the *miR-146* miRNA family, consisting of two evolutionary conserved miRNA genes: *miR-146a* and *miR-146b*. Previously, *miR-146a* was shown to be induced by endotoxin (lipopolysaccharide) through two consensus NF-kB-binding sites in the promoter region [23]. This region is highly homologous between human and mouse, suggesting an evolutionarily conserved regulatory mechanism for controlling *miR-146a* expression. A previous study provided some evidence that *miR-146a* overexpression in human glioblastomas exerts an anti-tumour effect, which is probably related to the capacity of this miRNA to target the 3′-UTR region of Notch 1 mRNA [24]. *miR-146a* constitutes an endogenous feedback system to counteract the oncogenic potential of dysregulated signalling pathways in human glioblastomas, such as activation of EGFR (endothelial growth factor receptor) and inactivation of Pten (phosphatase and tensin homolog) in gliomas. By regulating multiple targets, including the key NSC factor Notch 1, a *miR-146a*-mediated innate regulatory mechanism provides a native safeguarding mechanism to restrict the formation of glioma stem-like cells and glioma growth by directly controlling expression of Notch 1 [24]. Our current results demonstrate that *miR-146* plays an important role in mouse NSC differentiation and that Notch 1 is a key target of *miR-146* in mouse NSCs. Each miRNA could have multiple target genes [15,25]; indeed, several target genes have been predicted and some tested for *miR-146*, including those that encode the transcription factors NF-kB (nuclear factor kappa-light-chain-enhancer of activated B cells), BRMS1 (breast cancer metastasis-suppressor 1) and LPS (Lipopolysaccharides), as well as components of the FGF signalling pathway [26–28]. One of the questions addressed in the present study is whether the cell differentiation effect mediated by *miR-146* in NSCs is directly related to repression of Notch 1 expression. Results suggest that *miR-146* regulates NSC differentiation through repression of Notch 1 expression. Although Notch 1 is an important target gene of *miR-146*, other targets may also play a role in *miR-146* function in NSCs. Transfection of *miR-146* dramatically increases the percentage of GFAP-positive cells, and reduces the percentage of β-III-tubulin-positive cells *in vitro*. Previous studies have shown that *miR-146* overexpression represses cell proliferation in gliomas [24]. In the present study, we analysed the role of *miR-146* in neuronal differentiation from NSCs. Our results demonstrated that *miR-146* promotes differentiation of glial cells from NSCs and increases the percentage of GFAP-positive cells *in vitro*.

In the present study, we analysed the role of *miR-146* in proliferation and differentiation of NSCs. The results demonstrated that *miR-146* inhibited NSC proliferation and promoted differentiation of glial cells from NSCs and increased the percentage of β-III-tubulin-positive cells *in vitro* under contained serum medium conditions.

## Abbreviations

ddH_2_O: double distilled water
FGF: fibroblast growth factor
GFAP: Glial fibrillary acidic protein
HEK: human embryonic kidney
NF-kB: nuclear factor kappa-light-chain-enhancer of activated B cells
NSC: neural stem cell
Pax 6: paired box protein Pax-6; RBP-J, recombining binding protein suppressor of hairless; Sox2, sex determining region Y–box 2
